# Corrosion Behavior and Surface Characterization of Medium-Entropy Alloy Under Different Media Conditions

**DOI:** 10.3390/ma18050977

**Published:** 2025-02-22

**Authors:** Yingjie Zhang, Shuyang Ye, Qifan Min, Changlong Li, Delong Li, Bosheng Cao, Wensheng Ma, Kaimin Zhao, Yan Wang, Zhonghua Zhang

**Affiliations:** 1 School of Materials Science and Engineering, University of Jinan, Jinan 250022, China; 2 Key Laboratory for Liquid-Solid Structural Evolution and Processing of Materials, Ministry of Education, School of Materials Science and Engineering, Shandong University, Jinan 250061, China

**Keywords:** medium-entropy alloy, corrosion behavior, passive film, corrosion media, pitting, vacuum arc melting

## Abstract

The corrosion characteristics and passive behavior of as-cast Ni_40_Fe_30_Co_20_Al_10_ medium-entropy alloy (MEA) fabricated by the vacuum arc melting technique were investigated in 3.5 wt.% NaCl, 0.5 M HCl, and 0.5 M H_2_SO_4_ solutions. Although the impact of different solutions on the corrosion current density was not pronounced, the corrosion potential values of MEAs in H_2_SO_4_, HCl and NaCl solutions were −0.37, −0.58 and −1.16 V, respectively, indicating that the resistance to general corrosion in acidic solutions becomes strengthened. Through electrochemical passive region tests, surface morphology analysis and ICP testing, it was found that, due to the high-entropy effect and uniform single-phase structure, an optimized and stable passive film formed specifically in the Cl^−^-containing solution. The ion concentrations in the passive region of MEA in NaCl solution were an order of magnitude lower than those of other two samples, suggesting that its passive film formed exhibits a more prominent capacity to inhibit metal dissolution. Compared with electrochemical reactions in H_2_SO_4_ and HCl solutions, MEA shows enhanced pitting resistance in NaCl solution, which could be attributed to the presence of abundant unoxidized metal atoms (51.9 at.%). Al is identified as the primary component in the formation of the passive film, which plays a protective role for the Co-rich interior of the MEA. Although MEA has a relatively high passivation current in the H_2_SO_4_ solution, it has the widest passivation zone (1.87 V), indicating the optimized stability of the formed passive film. Moreover, it displays a high level of resistance to pitting corrosion in the solution containing only H^+^- and free of Cl^−^. Both the MEAs show significant grain-boundary corrosion in H_2_SO_4_ and HCl solutions. Among them, the MEA in HCl experiences more severe intragranular corrosion. Notably, MEA withstands the erosion of a single Cl^−^- or H^+^-containing solution, but it is unable to resist the synergistic effect of a solution containing both H^+^ and Cl^−^.

## 1. Introduction

Recently, high-entropy alloys (HEAs) and medium-entropy alloys (MEAs) have garnered considerable attention due to unique phase structures and outstanding performances. Specifically, their excellent mechanical properties [1,2,3,4] and outstanding corrosion resistance [5,6,7] have made them the focus of extensive research.

The inhomogeneity of dual-phase or multi-phase structures in HEAs leads to preferential corrosion of a certain phase regardless of the corrosion medium. This phenomenon is primarily attributed to the occurrence of micro-galvanic corrosion, as substantiated by prior research [8,9,10,11]. Conversely, the stable and uniform single-phase structure caused by high configurational entropy promoted unique corrosion resistance and afforded robust protection to the passive film in chloride-rich environments [12,13]. A single-phase MEA has a stable solid solution structure, which avoids the formation of micro-galvanic cells between different phases and has good corrosion resistance [14]. VNbTa MEA with a single BCC phase structure had better corrosion resistance than 316 L stainless steel in KOH solution [15].

Although Cr is one of the principal elements in classic HEAs/MEAs, conferring enhanced corrosion resistance [16,17,18], Cr-containing HEAs exhibited Cr segregation in inter-dendritic regions. These segregated areas are particularly susceptible to chloride ion attack [19,20]. Consequently, the customized design of corrosion-resistant HEAs/MEAs has emerged as a critical focus for practical applications. In previous work, we ascertained that the as-cast Cr-free Ni_40_Fe_30_Co_20_Al_10_ MEA manifested a homogeneous single-FCC structure, accompanied by satisfactory mechanical properties, thus heralding substantial application potential [21].

Owing to the absence of galvanic corrosion stemming from structural phase disparities, single-phase MEA/HEA exhibits heightened sensitivity to corrosive media. Moreover, presently, no comprehensive description exists for the overall corrosion resistance of single-phase alloys across diverse corrosive environments. Therefore, it is necessary to evaluate the corrosion behavior and pitting characteristics of single-FCC MEA across diverse media, which is also crucial for their application reliability. In the present study, the corrosion behavior and passive film characteristics of the as-cast MEA are investigated using electrochemical methods in three media of 3.5 wt.% NaCl (MEA-NaCl), 0.5 M HCl (MEA-HCl) and 0.5 M H_2_SO_4_ (MEA-H_2_SO_4_). This study aims to further explore the effects of individual chloride ions (Cl^−^), hydrogen ions (H^^+^^), as well as their synergistic interaction on the corrosion resistance of MEAs. Such an exploration holds profound significance for the prospective design of novel Cr-free MEAs possessing promising anti-corrosion performance and outstanding mechanical properties within complex media environments. Furthermore, it stands a good chance of emerging as a viable candidate material within the realms of oceanography and chemistry.

## 2. Materials and Methods

As-cast Ni_40_Fe_30_Co_20_Al_10_ MEA ingots with chemical homogeneity were prepared in a vacuum arc melting furnace using pure metals (99.9 wt.%). All samples for electrochemical tests were cut into 10 mm × 10 mm × 1 mm rectangular bars by using electrical discharge machining. The MEA surfaces were initially ground with 120 to 2000# SiC papers, followed by a mechanical polishing to produce a mirror-like finish. After that, the samples were rinsed in deionized water, ultrasonically cleaned in absolute alcohol, and finally dried in a vacuum drying oven.

All electrochemical measurements were completed on an electrochemical workstation (CHI660E). They were conducted in different solutions (3.5 wt.% NaCl, 0.5 M HCl and 0.5 M H_2_SO_4_) using platinum sheet (Tianjin Aida Hengsheng Technology Development Co., Ltd., Tianjin, China), Ag/AgCl electrode filled with saturated KCl solution and tested with MEA as the counter electrode, reference electrode and working electrode, respectively. Prior to the polarization measurement, the open circuit potential (OCP) was recorded for 30 min to reach a steady-state potential. Potentiodynamic polarization measurements were made at a scan rate of 3 mV/s. The electrochemical impedance spectroscopy (EIS) was carried out at the OCP with a sinusoidal potential amplitude of 5 mV, measured with a frequency range of 10^−2^~10^5^ Hz. Prior to EIS, both the working electrode and the platinum sheet electrode ought to be carefully polished and cleansed. Moreover, to confirm data reproducibility, the polarization tests were performed at least three times.

The morphologies and chemical compositions of the samples were obtained using field emission scanning electron microscopy (FESEM, ThermoFisher, Waltham, MA, USA, QUANTA FEG 250, FEI) operated in secondary electron mode combined with energy dispersive spectroscopy (EDS). The chemical compositions and valence states of the elements in the passive film were identified using X-ray photoelectron spectroscopy (XPS, ThermoFisher, Waltham, MA, USA, ESCALAB 250 Xi) analysis. XPS analysis was conducted on Thermo Scientific—Kα with a monochromatic Al-Kα X-ray radiation (1486.6 eV). The data obtained from XPS analysis were processed using Avantage software (Version 5.9921) and a standard C1s peak of 284.8 eV was used to calibrate the other peaks. The concentrations of various metal ions in the tested solutions were determined using inductively coupled plasma (ICP, ThermoFisher, Waltham, MA, USA, ICAP7200) spectroscopy.

## 3. Results and Discussion

**Figure 1:**
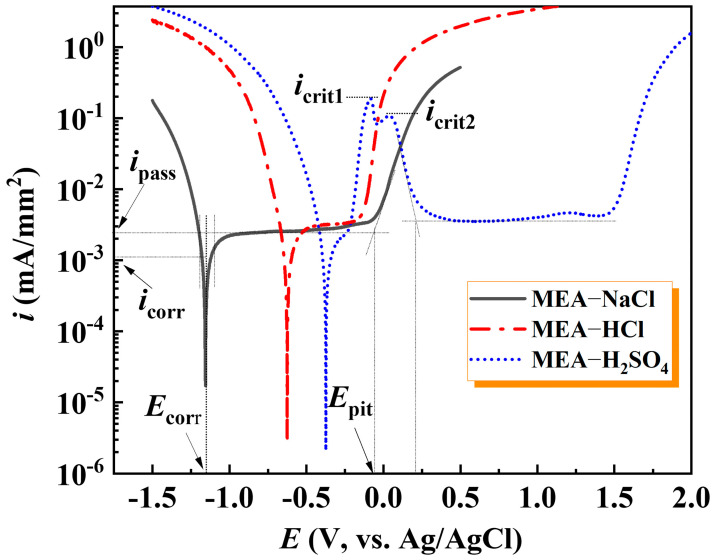
Potentiodynamic polarization curves of MEA under different corrosion media.

Figure 1 shows potentiodynamic polarization curves of MEA in diverse aqueous solutions, qualitatively assessing the effect of corrosive media on the corrosion resistance of the material itself. It is observable that the MEA-NaCl and MEA-HCl exhibit similar electrochemical corrosion responses, manifested as three typical stages of active dissolution, passivation and transpassivation. It indicates that the protective film is formed spontaneously at the corrosion potential. Unlike the other two solutions, the anodic polarization curve of MEA in H_2_SO_4_ solution also reveals an active-to-passive transition (pseudo-passivation), occurring (−0.34~0 V) prior to entering stable passivation zone. Notably, the characteristics of bimodal critical current density (*i*_crit_) are observed, showing the *i*_crit1_ and *i*_crit2_ measured at 0.19 and 0.10 mA/mm^2^, respectively. Similar complex transitions before the passivation of HEAs caused by H_2_SO_4_ solution have been reported [8,10,22,23]. It is possibly due to the rapid dissolution of the active region leading to an increase in current density. Subsequently, the progressively formed passive film impedes the overall dissolution rate.

Meanwhile, according to the fitting results of the polarization curves, the corresponding corrosion potential (*E*_corr_), passivation current density (*i*_pass_), pitting potential (*E*_pit_), corrosion current density (*i*_corr_) and passivation zone (Δ*E* = *E*_pit_ − *E*_corr_) are shown in Table 1. Because the experimental polarization curves do not exhibit linear Tafel regions [24], the *i*_corr_ was obtained after fitting in the region about ±60 mV of *E*_corr_ using the Butler–Volmer analysis (CHI660E software Version 15.03).

The impact of different solutions on the *i*_corr_ is not pronounced, but the *E*_corr_ values of MEA-H_2_SO_4_, MEA-HCl and MEA-NaCl are −0.37, −0.58 and −1.16 V, respectively, displaying that the resistance to general corrosion of MEA in acidic solutions becomes strengthened. A similar impact scenario has been reported that an increase in pH value promoted a negative shift in *E*_corr_ of CoCrFeNiMo HEA [25]. Thus, the smaller *i*_pass_ reflects a lower anodic dissolution rate, usually leading to a denser and more protective passive film being formed on the alloy surface [26]. The *i*_pass_ of MEA-NaCl is the smallest (2.50 × 10^−3^ mA/mm^2^), demonstrating that it is easiest to form a passive film under pure Cl^−^ medium. In addition, the *i*_pass_ of MEA-HCl (2.98 × 10^−3^ mA/mm^2^) is distinctly lower than that of the MEA-H_2_SO_4_ (3.84 × 10^−3^ mA/mm^2^). It shows more H^+^ ion solution is not conducive to the formation of passive film on MEA. However, the MEA-H_2_SO_4_ has widest ΔE (1.87 V), displaying high resistance to pitting in a solution containing only H^+^ and free of Cl^−^. However, Cl^−^ and H^+^ are inclined to combine with Al on the MEA-HCl surface to form metastable complexes [27], rationalizing the narrowest ΔE (0.43 V) in HCl solution.

**Figure 2:**
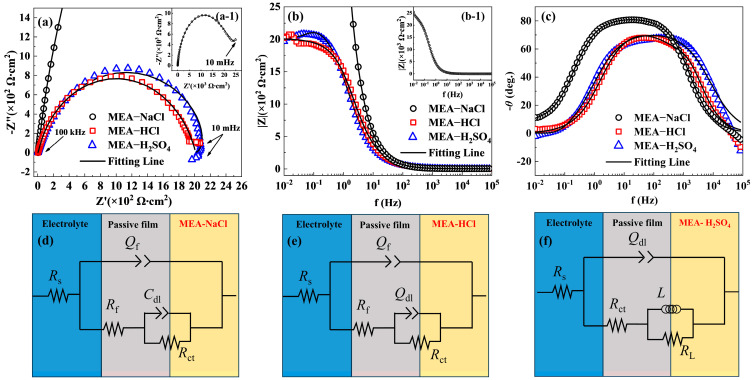
Electrochemical impedance plots of MEA subjected to different corrosion media: Nyquist plots (**a**) and whole Nyquist plot of MEA-NaCl alone (**a-1**), Bode modulus plots (**b**) and whole Bode modulus plot of MEA-NaCl alone (**b-1**), Bode phase angle plots (**c**), and equivalent circuits (**d**–**f**) for fitting EIS. (*R*_s_: solution resistance, *C*_dl_/*Q*_dl_: ideal/double-layer capacitance, *R*_ct_: charge transfer resistance, *R*_f_: passive film resistance, *Q*_f_: film capacitance, *L*: inductance, *R*_L_: inductance resistance).

It is generally accepted that the pitting resistance is closely correlated with the performance of passive films. Therefore, to further understand the corrosion mechanism of the MEA sample in different solutions, the EIS measurements were performed at the OCPs to explore the performance of the passive films. The Nyquist plots of the tested MEAs all exhibit a semicircle arc (Figure 2a), indicating that their corrosion reactions are dynamically controlled by charge transfer. Meanwhile, an inductive loop in the low-frequency region is observed for MEA in H_2_SO_4_ solution, which may be attributed to the charge transfer process and the relaxation process acquired by adsorbed intermediate products and the dissolution of the metal or oxide layer [27].

The diameter of the semicircle in the Nyquist plot is directly proportional to the polarization resistance of the sample. The larger the semicircle diameter of the sample, the greater the charge transfer resistance and the more stable and protective the oxide layer, indicating better corrosion resistance. The order of the semicircle diameters in the Nyquist plots is as follows: MEA-NaCl (massive) > MEA-H_2_SO_4_ > MEA-HCl; this suggests higher corrosion resistance of MEA-NaCl. The above results indicate the different electrochemical corrosion responses of MEA in three corrosion media. In general, the |Z| value corresponds to the polarization resistance of the material during corrosion at frequencies (*f*) between 0.01 and 0.1 Hz [28]. In the low-frequency region (0.01–0.1 Hz), the |Z| (polarization resistance, the approximation of the ohmic resistance at an infinite small frequency) of MEA-NaCl is significantly higher than that of MEA-H_2_SO_4_ and MEA-HCl, as shown in Figure 2b. These results are consistent with the variation trend of the Nyquist plots. This indicates the excellent corrosion resistance showing high passivation of MEA-NaCl. It can be attributed to the fact that a larger polarization resistance implies a decelerated electrochemical reaction rate [29]. Under normal circumstances, only a Bode phase angle close to 80° in the intermediate-frequency region proves that a stable passive film is formed on the surface of the material. MEA-NaCl has the largest phase angle (~80°) (Figure 2c), meaning better capacitive behavior and a more protective passive film [30]. It indicates the passive film formed on the MEA surface exhibits the strongest resistance to single-Cl^−^-ion-containing solution. MEA-HCl and MEA-H_2_SO_4_ have similar curves, revealing that their passive film mechanisms are the same.

The equivalent circuit models in Figure 2d,e utilized to fit the EIS data demonstrate that the electrode reactions of MEA-NaCl and MEA-HCl are controlled by the passive film and an ohmic drop, represented as similar circuits of *R*_s_ (*Q*_f_ (*R*_f_ (*C*_dl_*R*_ct_))) and *R*_s_ (*Q*_f_ (*R*_f_ (*Q*_dl_*R*_ct_))), respectively. The parameter *Q* is usually adopted instead of C to represent the capacitive behavior due to the surface heterogeneity [31]. Due to the increase in surface roughness caused by H^+^, *Q*_dl_ is employed instead of *C*_dl_ in MEA-HCl. MEA-H_2_SO_4_ is represented as *R*_s_ (*Q*_dl_ (*R*_ct_ (*LR*_L_), where *L* is correlated to the adsorption/desorption of ions and molecules at the interface between the electrolyte and substrate [32], as presented in Figure 2f. The fitting results are shown in Table 2. The MEA-NaCl has the highest values of *R*_ct_ (5051 Ω cm^2^) and *R*_f_ (21940 Ω cm^2^), which are approximately 2.5 and 562 times greater than those of MEA-HCl. It indicates that the passive film formed in a solution containing only Cl^−^ significantly impedes charge transfer and exhibits a slow dissolution rate, suggesting that it offers the best protective capability for MEA. The value of *Q*_dl_ obtained for the MEA-H_2_SO_4_ is higher than that of the MEA-HCl, suggesting the higher compactness of its passive film. From the parameters of the equivalent circuit, the large total resistance *R*_t_ (=*R*_f_ + *R*_ct_) of MEA implies enhanced stability of the passive film. The large total resistance *R*_t_ of MEA-NaCl (26991 Ω cm^2^) is considerably larger than that of MEA-H_2_SO_4_ (2026 Ω cm^2^) and MEA-HCl (2010.59 Ω cm^2^), thereby demonstrating the superior protection of its passive film.

The thickness of the passive film formed on the HEA surface under various solution conditions was estimated in accordance with the power law model [33]. The effective capacitance of the passive film can be calculated by Equation (1) [34].

*C*_eff_ = *gQ* (*εε*_0_*ρ*_d_)^1−*n*^ (1)

where *C*_eff_ represents effective capacitance of the passive film, *g* = 1 + 2.88 (1 − *n*)^2.375^, *ρ*_d_ = 500 Ω cm, *ε* is the dielectric constant of the passive film (15.6 as used in [35,36]), and *ε*_0_ is the vacuum permittivity constant (*ε*_0_ = 8.854 × 10^−14^ F cm^−1^).

The passive film thickness (*d*) can be calculated by Equation (2).

(2) $$d=\frac{\varepsilon \varepsilon_{0}}{C_{\mathrm{eff}}}$$

*d* is the passive film thickness, and the calculated values of *d* according to Equations (1) and (2) are shown in Table 2. The *d* of the MEA-NaCl (2.93 nm) is thicker than that of the other two samples, which is consistent with EIS analysis. As the concentration of H^+^ increases, the *d* becomes significantly decreased. However, according to the EIS results, the corrosion resistance of MEA-H_2_SO_4_ is optimized compared to MEA-HCl. It has been reported that the improvement of corrosion resistance of the passive film depended not only on the film thickness change, but was related to the composition change of the passive film [37]. Enhancing the protective ability of the FeCoCrNiMo alloy in the highly aggressive Cl^−^ environment hinges upon modulating the composition and thickness of the passive film [38]. Consequently, it is imperative that the chemical states and elemental contents in the surface film are subjected to further investigation.

**Figure 3:**
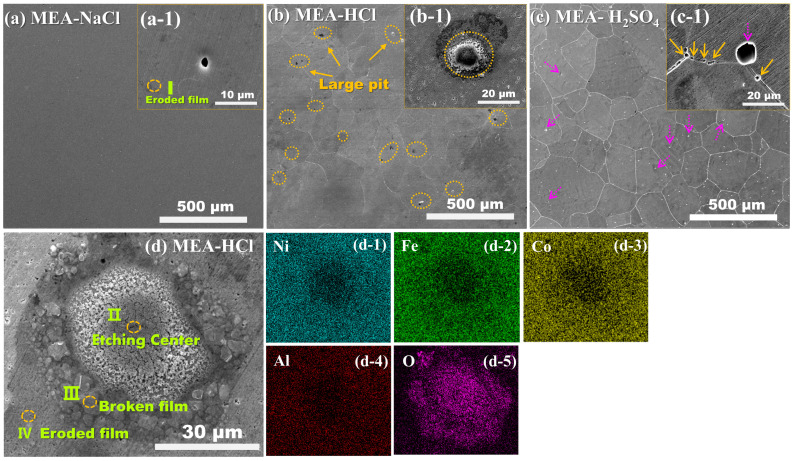
FESEM micrographs of MEA after corrosion in 3.5 wt.% NaCl (**a**), 0.5 M HCl (**b**) and 0.5 M H_2_SO_4_ (**c**) solutions and corresponding insets of corrosion pit morphologies ((**a-1**), (**b-1**) and (**c-1**), *E*_pit_ is the cut-off potential). Zoom-in view of pits in MEA-HCl (**d**) and the corresponding elemental mapping images (**d-1**–**d-5**).

Figure 3 shows the corrosion morphologies of the MEA in three corrosion media. Except for the tiny pit speculated to be a casting defect (~2 μm) (Figure 3a-1), the MEA-NaCl exhibits a smooth and compact passive film (Figure 3a). In Region I, Al and oxygen (O) are pronouncedly enriched, and elements of Fe and Ni are relatively enriched, while Co is relatively scarce (Table 3). This selective dissolution of Co to a certain extent that occurs during the surface passivation on the MEA in NaCl solution is beneficial for the enrichment of Al in the passive film. In combination with Ni and Fe, Al shows a prominent contribution in promoting passive film formation. Typical grain-boundary corrosion characteristics are found for MEA-HCl (Figure 3b) and MEA-H_2_SO_4_ (Figure 3c). On the basis of the zoomed-in view in Figure 3b-1, MEA-HCl unveils severe intragranular corrosion, characterized by numerous and densely distributed small pits of ~1 μm and scattered large pits of ~20 μm (as marked by dash circles) within grains. In Figure 3c,c-1, pits with lower distribution density in MEA-H_2_SO_4_ are distributed on grain boundaries (~3 μm) and inside grains (~10 μm), as marked by solid and dashed arrows, respectively. However, the distribution density of surface pits is much lower than that in the MEA-HCl. These pits at two different locations in MEA-H_2_SO_4_ are promoted by the pseudo-passivation phenomenon along the grain boundaries and then propagate into the grains. Based on the observation and comparison of corrosion morphologies of three samples, it is demonstrated that more H^+^ ions (MEA-H_2_SO_4_) exhibit a more pronounced attacking effect on grain boundaries. The synergistic effect of H^+^ and Cl^−^ causes more significant damage to passive film on the grain surface.

**Figure 4:**
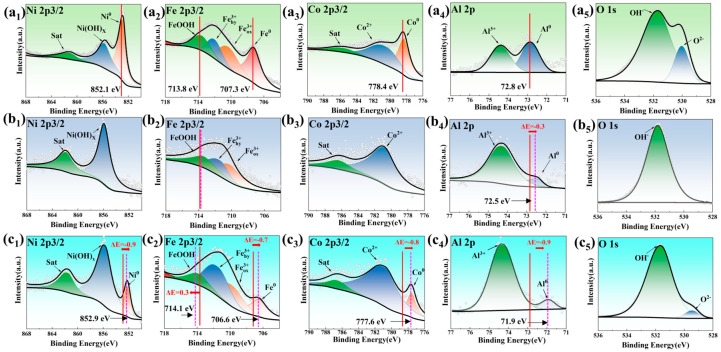
XPS spectra of corroded surfaces of MEA-NaCl (**a_1_**–**a_5_**), MEA-HCl (**b_1_**–**b_5_**), and MEA-H_2_SO_4_ (**c_1_**–**c_5_**).

**Figure 5:**
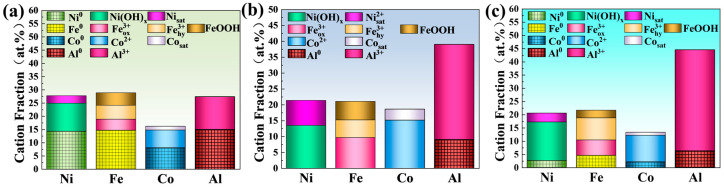
Cationic and metallic state fractions of MEA-NaCl (**a**), MEA-HCl (**b**) and MEA-H_2_SO_4_ (**c**) obtained from XPS results.

Figure 3d shows the complex morphology of a large pit with internal etching (Region II) and peripheral accumulation of ruptured passive film (or corrosion product) (Region III) in MEA-HCl. Fragmented corrosion products accumulate around the corrosion pit. According to elemental mappings (Figure 3d-1–d-5) and FESEM-EDS results (Table 3), the oxygen and Cl contents become decreased from the pit center (Region II) to the adjacent passive film (Region IV), and the presence of Cl outside the pit is not detected. According to the nominal component ratio of each principal element in MEA-HCl, the pit center (Region II) is rich in Co and poor in Al, Ni and Fe. Moreover, the Al content shows a pronounced increase of 62% (from Region II to III) and 127% (from Region III to IV). Combined with the component distribution in Region I, it is further demonstrated that Al is the main component to form the passive film, protecting the Co-rich interior [27].

Importantly, the corrosion resistance of alloys in a certain corrosion environment depends on the composition of the passive film. To investigate the passive films on the surface, the samples were charged under potentiostatic conditions for 4 h at a chosen potential (−0.5 V for 3.5 wt.% NaCl, −0.6 V for 0.5 M HCl and 1.0 V for 0.5 M H_2_SO_4_) to produce the stable passive films. The compositions of the passive films were subsequently analyzed by XPS, as revealed in Figure 4. The peak position calibration was performed with reference to the C 1s (around 284.8 eV for three samples), as shown in Figure S1. The spectra of Ni 2p3/2, Fe 2p3/2, Co 2p3/2 and Al 2p are presented. The components of MEA-NaCl are mainly metallic states of Ni (Ni^0^), Fe (Fe^0^), Co (Co^0^) and Al (Al^0^), and a certain amount of Co/Al/Fe oxides and Fe/Ni hydroxides (Figure 5a). The abundant metallic states are caused by the fact that the principal elements are not readily oxidized in NaCl solution. However, in only Cl^−^-containing solution, the stable and dense passive film is still formed under weak oxidation conditions due to entropy effect and uniform single-phase structure. Although containing metallic states, the oxidation states in MEA-H_2_SO_4_ play a prominent role (Figure 5c), and the peaks of metallic states shift to lower binding energies (Figure 4c_1_–c_4_), implying a higher electron density [39]. However, except for Al (Al^0^), other metallic states are not found in the MEA-HCl (Figure 5b), revealing the high oxidizability of a combination of H^+^ and Cl^−^.

The Ni_x_^+^ ion peaks are the same for these three samples (Figure 4a_1_–c_1_). Compared with MEA-NaCl and MEA-HCl, the Fe^3+^ ion peak in MEA-H_2_SO_4_ exhibits positive shifts of 0.3 eV (Figure 4c_2_), promoting the binding of Fe atoms with -OOH groups on the surface in the solution with more H^+^ ions (H_2_SO_4_), thus facilitating the formation of hydroxides. Figure 4a_5_,c_5_ depict two constituent peaks of O 1s peaks in MEA-NaCl and MEA-H_2_SO_4_, corresponding to metal oxides of O^2−^ and hydroxides of OH^−^. Differently, the concentration of OH^−^ ions in the MEA-H_2_SO_4_ is significantly higher than that of O^2−^ ions, resulting in an alkaline environment after electrochemical reaction in acidic media. Specifically, only hydroxides of OH^−^ exist in the MEA-HCl (Figure 4b_5_), demonstrating that the combination of H^+^ and Cl^−^ leads to the hydroxides forming the bulk of the passive film.

On the basis of XPS analysis results, the fractions of cationic and metallic states of the principal elements within the passive film under different media were calculated and are illustrated in Figure 5. The passive film components of MEA-NaCl predominantly consist of the metallic states of Ni (14.3 at.%), Fe (14.7 at.%), Co (8.0 at.%) and Al (14.9 at.%), along with a certain quantity of oxides and hydroxides, as presented in Figure 5a. Notably, while the MEA-H_2_SO_4_ and MEA-HCl exhibit a certain amount of four zerovalent metal states (15.5 at.%) and only Al^0^ state (9.0 at.%), respectively, there is no significant disparity in the main productions of the passive films formed in the two acidic media (HCl and H_2_SO_4_), with more hydroxides being observed.

It has been reported that the atoms of unoxidized metals in the passive film of AlTiVCr HEA were a considerable factor to be responsible for the good corrosion resistance of HEAs [40], preventing the injection and ejection of metal in the film. The point defect model suggests that the point defects facilitate the current transport through the metallic passive film, leading the participation in the reactions at the metal/film and film/solution interfaces, which determines the passivation and breakdown of the passive film [16]. Based on the point defect model, the proposed corrosion mechanism of HEA indicates that the atoms of unoxidized metal hinder the transport of point defects through the film, forming a barrier at the interfaces, thereby improving the corrosion resistance of HEA [40]. Therefore, given the plenitude of unoxidized metal atoms on the film surface, this furnishes a reliable mechanistic explanation for the improved passivity with the high pitting resistance of MEA-NaCl.

Note that the content of Co and its related reactants is considerably low in all three media, suggesting that Co plays a negligible role in facilitating the passive film formation and tends to reside in the unreacted interior or is selectively dissolved.

To better understand the corrosion process of Ni_40_Fe_30_Co_20_Al_10_ MEA in three distinct solutions, ICP tests were performed to ascertain the concentration of metal ions in the solutions, as presented in Table 4. Unlike the element dissolution rates which are sluggish in passive region and accelerate in the transpassive region for the other two samples, a high amount of Al ions (57.70 mg/L) from MEA-HCl dissolve during the passive stage. The passive film of MEA-HCl fails to effectively impede the continuous dissolution of Al, thus showing selective dissolution during passivation. Evidently, compared with the other two samples, the ion concentrations of MEA-NaCl in the passive region are an order of magnitude lower, indicating the formation of a highly stable passive film that is robust enough to hinder the dissolutions of metal elements. It should be noted that the MEA-HCl shows a marginally elevated metal dissolution rate in the passive stage compared to MEA-H_2_SO_4_. It suggests that the combination of H^+^ and Cl^−^ degrades the passive film quality and diminishes its protective capacity for the internal unreacted layer, which is further corroborated by a large number of densely packed pits on the surface (Figure 3b). Evidently, the ion concentrations in the passive region of MEA-NaCl are one order of magnitude lower than those of other two samples. Moreover, the increment in the leaching amount of Al ions in the transpassive region, relative to the passive region, is substantially lower. It displays that the optimized and stable passive film formed on the MEA-NaCl is potent enough to prevent metal dissolution. Consequently, Al within MEA serves to promote the passive film formation and further stabilizes it, achieving a dual contribution to the pitting resistance improvement.

## 4. Conclusions

In this study, the characteristics of the passive films formed on the MEA under three corrosion media materials are investigated by employing a combination of electrochemical polarization curve, FESEM, EIS and XPS testing methods. Although the impact of different solutions on the corrosion current density is not pronounced, the *E*_corr_ values of MEAs in H_2_SO_4_, HCl and NaCl solutions are −0.37, −0.58 and −1.16 V, respectively, indicating that its resistance to general corrosion in acidic solutions is more advantageous. The comprehensive experimental results demonstrate that the passive film generated on the MEA-NaCl exhibits enhanced stability and superior anti-corrosion features compared to those formed in H_2_SO_4_ and HCl solutions. The surface morphology at the cut-off potential demonstrates that both MEA-H_2_SO_4_ and MEA-HCl suffer from significant grain-boundary corrosion. The HCl actually causes more severe damage to the intragranular structure of MEA-HCl. XPS data further depict that the abundance of unoxidized metal atoms (51.9 at.%) present on the surface of the passive film plays a crucial role in enhancing the pitting resistance of MEA-NaCl. Moreover, Al achieves a dual contribution to the improvement of pitting resistance by facilitating the formation of the passive film and subsequently stabilizing it. The ICP result shows that the ion concentrations in the passive region of MEA-NaCl are an order of magnitude lower than those of the other two samples. This finding clearly suggests that the passive film formed on the MEA-NaCl possesses a more prominent capacity to inhibit metal dissolution. Consequently, MEA demonstrates excellent resistance to erosion when exposed to solutions containing either single Cl^−^ or H^+^ ions. 

## Figures and Tables

**Table 1:** Electrochemical parameters derived from potentiodynamic polarization curves of Ni_40_Fe_30_Co_20_Al_10_ MEA in different corrosion solutions.

Samples	*E*_corr_ (V, vs. Ag/AgCl)	*E*_pit_ (V, vs. Ag/AgCl)	∆*E* (V, vs. Ag/AgCl)	*i*_corr_ × 10^−4^ (mA/mm^2^)	*i*_pass_ × 10^−3^ (mA/mm^2^)
MEA-NaCl	−1.16	−0.07	1.09	13.99	2.50
MEA-HCl	−0.58	−0.15	0.43	14.73	2.98
MEA-H_2_SO_4_	−0.37	1.50	1.87	15.35	3.84

**Table 2:** Fitting parameters obtained from electrochemical impedance curves of Ni_40_Fe_30_Co_20_Al_10_ MEAs in 3.5 wt.% NaCl, 0.5 M HCl and 0.5 M H_2_SO_4_ solutions. (The dashes (“-”) in table denote “not applicable”).

Samples	*R*_s_ (Ω cm^2^)	*Q*_f_ × 10^−5^ (Ω^−1^ cm^−2^ S^n^)	n_1_	*R*_f_ (Ω cm^2^)	*R*_ct_ (Ω cm^2^)	*Q*_dl_ × 10^−6^ (Ω^−1^ cm^−2^ S^n^)	n_2_	*L* (H)	*R*_L_ (Ω cm^2^)	*C*_dl_ × 10^−3^ (F cm^−2^)	*d* (nm)
MEA-NaCl	5.03	1.88	0.90	21,940	5051	-	-	-	-	1.92	2.93
MEA-HCl	3.83	1.35	0.95	38.59	1972	4.33	0.77	-	-	-	2.37
MEA-H_2_SO_4_	1.06	-	-	-	2026	11.1	0.81	604	249.7	-	1.58

**Table 3:** EDS results of different regions of corroded MEA obtained from Figure 3a,d.

Regions	Ni	Fe	Co	Al	O	Cl
Nominal Component (at.%)	40	30	20	10	-	-
I	34.17	24.32	14.01	11.69	15.81	-
II	18.89	14.84	12.03	3.24	39.88	11.12
III	25.58	21.31	14.71	5.27	27.97	5.16
IV	33.63	28.20	18.29	7.37	12.51	-

**Table 4:** Concentration of metal ions after electrochemical corrosion.

Samples	Ni (mg/L)		Fe (mg/L)		Co (mg/L)		Al (mg/L)	
	Passive Region	Transpassive Region	Passive Region	Transpassive Region	Passive Region	Transpassive Region	Passive Region	Transpassive Region
MEA-NaCl	0.77	53.23	0.26	48.44	0.71	47.55	2.50	13.85
MEA-HCl	3.95	22.80	6.10	25.68	3.48	19.98	57.70	19.31
MEA-H_2_SO_4_	2.37	112.98	3.57	119.16	2.27	100.57	24.60	65.55

## Data Availability

The original contributions presented in this study are included in the article and Supplementary Material. Further inquiries can be directed to the corresponding author.

## Supplementary Materials

The following supporting information can be downloaded at: https://www.mdpi.com/article/10.3390/ma18050977/s1, Figure S1: XPS full spectra of corroded surfaces of MEA-NaCl (**a**), MEA-HCl (**b**), and MEA-H_2_SO_4_ (**c**).

## References

[1] Shi P.J., Li R.G., Li Y., Zhong Y.B., Ren W.L., Shen Z., Zheng T.X., Peng J.C., Liang X., Hu P.F. (2021). Hierarchical crack buffering triples ductility in eutectic herringbone high-entropy alloys. Science.

[2] Yuan J.P., Yang Y.J., Duan S.G., Dong Y., Li C.Q., Zhang Z.R. (2023). Rapid Design, Microstructures, and Properties of Low-Cost Co-Free Al-Cr-Fe-Ni Eutectic Medium Entropy Alloys. Materials.

[3] Wang Y.F., Ma X.L., Guo F.J., Zhao Z.F., Huang C.X., Zhu Y.T., Wei Y.G. (2023). Strong and ductile CrCoNi medium-entropy alloy via dispersed heterostructure. Mater. Des..

[4] Ma Y., Yuan F.P., Yang M.X., Jiang P., Ma E., Wu X.L. (2018). Dynamic shear deformation of a CrCoNi medium-entropy alloy with heterogeneous grain structures. Acta Mater..

[5] Shuang S., Lyu G.J., Chung D., Wang X.Z., Gao X., Mao H.H., Li W.P., He Q.F., Guo B.S., Zhong X.Y. (2023). Unusually high corrosion resistance in Mo_x_CrNiCo medium entropy alloy enhanced by acidity in aqueous solution. J. Mater. Sci. Technol..

[6] Wetzel A., Au M., Dietrich P.M., Radnik J., Ozcan O., Witt J. (2022). The comparison of the corrosion behavior of the CrCoNi medium entropy alloy and CrMnFeCoNi high entropy alloy. Appl. Surf. Sci..

[7] Wang D.P., Meng H., Wang J.B., Wang Z.J., Ye Y., Dong Z.Z., Wu Y.C., Wang Y.X. (2024). Corrosion performance of a high-strength FeNiCrAl medium-entropy alloy compared with 304 stainless steel in KOH solution. Appl. Surf. Sci..

[8] Wan X.L., Lan A.D., Zhang M., Jin X., Yang H.J., Qiao J.W. (2023). Corrosion and passive behavior of Al_0.8_CrFeNi_2.2_ eutectic high entropy alloy in different media. J. Alloys Compd..

[9] Cui P.C., Bao Z.J., Liu Y., Zhou F., Lai Z.H., Zhou Y., Zhu J.C. (2022). Corrosion behavior and mechanism of dual phase Fe_1.125_Ni_1.06_CrAl high entropy alloy. Corros. Sci..

[10] Yen C.C., Lu H.N., Tsai M.H., Wu B.W., Lo Y.C., Wang C.C., Chang S.Y., Yen S.K. (2019). Corrosion mechanism of annealed equiatomic AlCoCrFeNi tri-phase high entropy alloy in 0.5 M H_2_SO_4_ aerated aqueous solution. Corros. Sci..

[11] Shi Y.Z., Yang B., Xie X., Brechtl J., Dahmen K.A., Liaw P.K. (2017). Corrosion of Al_x_CoCrFeNi high-entropy alloys: Al-content and potential scan-rate dependent pitting behavior. Corros. Sci..

[12] Li M.J., Chen Q.J., Cui X., Peng X.Y., Huang G.S. (2021). Evaluation of corrosion resistance of the single-phase light refractory high entropy alloy TiCrVNb_0.5_Al_0.5_ in chloride environment. J. Alloys Compd..

[13] Sünbül S.E., İçïn K., Şeren F.Z., Şahin Ö., Çakil D.D., Sezer R., Öztürk S. (2021). Determination of structural, tribological, isothermal oxidation and corrosion properties of Al–Co–Cr–Fe–Ni–Ti–Cu high-entropy alloy. Vacuum.

[14] Liu J.J., Zhao Y.C., Hu R.N., Zhang M.Y., Ding Y.T. (2023). Effects of Cu and Ag elements on corrosion resistance of Dual-Phase Fe-Based medium-entropy alloys. Materials.

[15] Han Z.H., Guo C.H., Huang C.D., Fan X.Y., Zhang J.Y., Liu G., Wang H.Y., Wei R. (2024). Corrosion resistant body-centered cubic VNbTa refractory medium-entropy alloy. Corros. Sci..

[16] Fu Y., Li J., Luo H., Du C.W., Li X.G. (2021). Recent advances on environmental corrosion behavior and mechanism of high-entropy alloys. J. Mater. Sci. Technol..

[17] Yan X.L., Guo H., Yang W., Pang S.J., Wang Q., Liu Y., Liaw P.K., Zhang T. (2021). Al_0.3_CrxFeCoNi high-entropy alloys with high corrosion resistance and good mechanical properties. J. Alloys Compd..

[18] Guo Z.H., Liu M., Ma Y., Yu H., Jing S.R., Yan Y. (2025). Hydrogen embrittlement and corrosion resistance of NiCoCr-based equimolar face-centered cubic medium-/high-entropy alloys. Corros. Sci..

[19] Chai W.K., Lu T., Pan Y. (2020). Corrosion behaviors of FeCoNiCr*_x_*(*x* = 0, 0.5, 1.0) multi-principal element alloys: Role of Cr-induced segregation. Intermetallics.

[20] Tsau C.H., Lin S.X., Fang C.H. (2017). Microstructures and corrosion behaviors of FeCoNi and CrFeCoNi equimolar alloys. Mater. Chem. Phys..

[21] Wang N.R., Zhang Y.J., Cai L., Huang Q.K., Zhang Z.Y., Ma W.S., Wu H., Wang Y. (2024). Tailoring recrystallization for optimum mechanical combination in Ni-rich medium-entropy alloy via simplified thermomechanical treatment. J. Alloys Compd..

[22] Yao Y.H., Jin Y.H., Gao W., Liang X.Y., Chen J., Zhu S.D. (2021). Corrosion behavior of AlFeCrCoNiZr_x_ high-entropy alloys in 0.5 M sulfuric acid solution. Metals.

[23] Fu Y., Luo H., Chen X.R., Prabhakar J.M., Wang X.F., Cheng H.X., Du C.W., Hu S.Q., Li X.G. (2024). The corrosion behavior and passive film properties of the cast and annealed AlCoCrFeNi_2.1_ eutectic high-entropy alloy in sulfuric acid solution. Corros. Sci..

[24] Flitt H.J., Schweinsberg D.P. (2005). Evaluation of corrosion rate from polarisation curves not exhibiting a Tafel region. Corros. Sci..

[25] Wang Z., Zhang G.H., Fan X.H., Jin J., Zhang L., Du Y.X. (2022). Corrosion behavior and surface characterization of an equiatomic CoCrFeMoNi high-entropy alloy under various pH conditions. J. Alloys Compd..

[26] Gao M.H., Zhang S.D., Yang B.J., Wang J.Q. (2018). Influence of yttrium on surface chemistry and stability of passive film in Al based binary metallic glasses. Appl. Surf. Sci..

[27] Fu Y., Dai C.D., Luo H., Li D.Y., Du C.W., Li X.G. (2021). The corrosion behavior and film properties of Al-containing high-entropy alloys in acidic solutions. Appl. Surf. Sci..

[28] Ha H.Y., Jang M.H., Lee T.H. (2016). Influences of Mn in solid solution on the pitting corrosion behaviour of Fe-23 wt%Cr-based alloys. Electrochim. Acta..

[29] Marcelin S., Pébère N., Régnier S. (2013). Electrochemical characterisation of a martensitic stainless steel in a neutral chloride solution. Electrochim. Acta..

[30] Ray M., Singh V.B. (2011). Effect of sulfuric acid on corrosion and passivation of 316 SS in organic solution. J. Electrochem. Soc..

[31] Mert B.D., Yüce A.O., Kardas G., Yazıcı B. (2014). Inhibition effect of 2-amino-4-methylpyridine on mild steel corrosion: Experimental and theoretical investigation. Corros. Sci..

[32] Hsu K.M., Chen S.H., Lin C.S. (2021). Microstructure and corrosion behavior of FeCrNiCoMn_x_ (x = 1.0, 0.6, 0.3, 0) high entropy alloys in 0.5 M H_2_SO_4_. Corros. Sci..

[33] Musiani M., Orazem M.E., Pébère N., Tribollet B., Vivier V. (2011). Constant-Phase-Element behavior caused by coupled resistivity and permittivity distributions in films. J. Electrochem. Soc..

[34] Orazem M.E., Frateur I., Tribollet B., Vivier V., Marcelin S., Pebere N., Bunge A.L., White E.A., Riemer D.P., Musiani M. (2013). Dielectric properties of materials showing constant-phase-element (CPE) impedance response. J. Electrochem. Soc..

[35] Wang Z., Feng Z., Zhang L. (2020). Effect of high temperature on the corrosion behavior and passive film composition of 316 L stainless steel in high H_2_S-containing environments. Corros. Sci..

[36] Wang Y., Jin J., Zhang M., Wang X., Gong P., Zhang J., Liu J. (2021). Effect of the grain size on the corrosion behavior of CoCrFeMnNi HEAs in a 0.5 M H_2_SO_4_ solution. J. Alloys Compd..

[37] Xing B.W., Ding Q., Jin B.Q., Zuo X.J., Zhang N.N., Yin S. (2022). Corrosion resistance and passivation behavior of CoCrFeNi-TiAl high-entropy alloy coatings in acidic solutions. J. Therm Spray Tech..

[38] Dai C.D., Zhao T.L., Du C.W., Liu Z.Y., Zhang D.W. (2020). Effect of molybdenum content on the microstructure and corrosion behavior of FeCoCrNiMox high-entropy alloys. J. Mater. Sci. Technol..

[39] He R., Yang L., Zhang Y., Jiang D.C., Lee S.H., Horta S., Liang Z.F., Lu X., Moghaddam A.O., Li J.S. (2023). A 3d-4d-5d high entropy alloy as a bifunctional oxygen catalyst for robust aqueous Zinc–Air batteries. Adv. Mater..

[40] Qiu Y., Thomas S., Gibson M.A., Fraser H.L., Pohl K., Birbilis N. (2018). Microstructure and corrosion properties of the low-density single-phase compositionally complex alloy AlTiVCr. Corros. Sci..

