# Supplementary material for: Corrosion Behavior and Surface Characterization of Medium-Entropy Alloy Under Different Media Conditions
Source: Materials (Basel). 2025 Feb 22;18(5):977. doi: 10.3390/ma18050977 (PMC11901315; doi:10.3390/ma18050977)
Supplement: Supplementary file 1 [file materials-18-00977-s001.zip › materials-3455909-supplementary.pdf]

Corrosion Behavior and Surface Characterization of Medium-Entropy Alloy Under Different Media Conditions

Yingjie Zhang [a], Shuyang Ye [a], Qifan Min [a], Changlong Li [a], Delong Li [a], Bosheng Cao [a], Wensheng Ma [a], Kaimin Zhao [a], Yan Wang [a,*], Zhonghua Zhang [b,*]

[a] School of Materials Science and Engineering, University of Jinan, Jinan 250022, China

[b] Key Laboratory for Liquid-Solid Structural Evolution and Processing of Materials (Ministry of Education), School of Materials Science and Engineering, Shandong University, Jinan 250061, China

* Corresponding author. E-mail address: zh_zhang@sdu.edu.cn (Z.Z.) and mse_wangy@ujn.edu.cn (Y.W.)

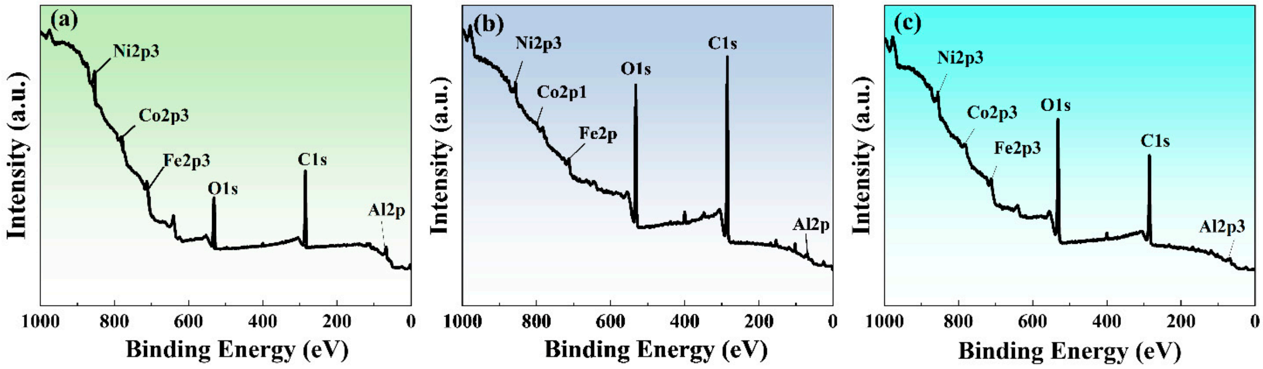

Figure S1. XPS full spectra of corroded surfaces of MEA-NaCl (**a**), MEA-HCl (**b**), and MEA-H_2SO_4 (**c**).
